# Nanocomposite-Based Microstructured Piezoresistive Pressure Sensors for Low-Pressure Measurement Range

**DOI:** 10.3390/mi9020043

**Published:** 2018-01-26

**Authors:** Vasileios Mitrakos, Philip J. W. Hands, Gerard Cummins, Lisa Macintyre, Fiona C. Denison, David Flynn, Marc P. Y. Desmulliez

**Affiliations:** 1 Multimodal Sensing and Micro-Manipulation Centre, School of Engineering & Physical Sciences, Heriot-Watt University, Edinburgh EH14 4AS, UK; g.cummins@hw.ac.uk (G.C.); m.desmulliez@hw.ac.uk (M.P.Y.D.); 2 Institute for Integrated Micro & Nano Systems (IMNS), School of Engineering, University of Edinburgh, Edinburgh EH9 3FF, UK; philip.hands@ed.ac.uk; 3 School of Textiles & Design, Heriot-Watt University, Galashiels TD7 4LF, UK; l.m.macintyre@hw.ac.uk; 4 Queen’s Medical Research Institute, MRC Centre for Reproductive Health, University of Edinburgh, Edinburgh EH16 4TJ, UK; fiona.denison@ed.ac.uk; 5 Smart Systems Group, School of Engineering & Physical Sciences, Heriot-Watt University, Edinburgh EH14 4AS, UK; d.flynn@hw.ac.uk

**Keywords:** pressure sensor, piezoresistive sensor, carbon nanotubes, quantum tunneling composite

## Abstract

Piezoresistive pressure sensors capable of detecting ranges of low compressive stresses have been successfully fabricated and characterised. The 5.5 × 5 × 1.6 mm^3^ sensors consist of a planar aluminium top electrode and a microstructured bottom electrode containing a two-by-two array of truncated pyramids with a piezoresistive composite layer sandwiched in-between. The responses of two different piezocomposite materials, a Multiwalled Carbon Nanotube (MWCNT)-elastomer composite and a Quantum Tunneling Composite (QTC), have been characterised as a function of applied pressure and effective contact area. The MWCNT piezoresistive composite-based sensor was able to detect pressures as low as 200 kPa. The QTC-based sensor was capable of detecting pressures as low as 50 kPa depending on the contact area of the bottom electrode. Such sensors could find useful applications requiring the detection of small compressive loads such as those encountered in haptic sensing or robotics.

## 1. Introduction

In recent years an increasing variety of non-commercial contact pressure sensors based on flexible elastomeric materials have been reported. These sensors are a response to a growing need to measure low pressure loads across a range of applications, including but not limited to healthcare monitoring in wearable applications, haptic sensing or robotic touch applications [1,2,3,4,5]. The various pressure or force transduction mechanisms used in these sensors range from piezoresistive [6], piezoelectric [7], triboelectric [8] to capacitive sensing [9,10,11] amongst others. 

Out of the aforementioned pressure sensors, those that rely on a piezoresistive force-sensitive response are the preferred choices for low-cost applications, due to the reduced complexity in the required readout electronics and manufacturing process. These piezoresistive materials can be compact since they generally consist of uniformly dispersed conductive particles within an elastomeric material. 

Piezoresistive sensors based on conductive elastomeric composites, such as carbon nanotubes (CNT)-Polydimethylosiloxane (PDMS) composites, usually exhibit their piezoresistive sensitivity to compressive levels above the megaPascal (MPa) threshold [12,13,14], a performance that is deemed inadequate for the majority of the aforementioned applications. A variety of methods have been used to obtain improved piezoresistive sensitivity in lower pressure regimes. These include (1) microstructuring of the composite surface, such as carbon nanotubes-polydimethylsiloxane layers (CNT-PDMS) with pyramids or pillars and placing two such films in an interlocked configuration [15,16]; (2) embedding of CNT films on a PDMS diaphragm [17,18] or CNT fillers in a bilayer pillar PDMS structure [19]. The primary disadvantage of these methods is the increase in manufacturing complexity and cost. 

This article describes a simple method of improving the resolution of piezoresistive pressure sensors to lower pressures, which involves micro-structuring the bias electrodes of the sensor to form truncated pyramid structures. These structures increase the effective contact area to the piezoresistive film. Two different piezoresistive composite films, with a positive and a negative piezoresistive response respectively, were evaluated with this method: an in-house developed Multi-Walled Carbon Nanotube (MWCNT)-PDMS piezoresistive composite and a commercially available Quantum Tunnelling Composite (QTC). 

The above piezoresistive films were chosen to enable a quantifiable piezoresistive response to low compressive stimuli described in detail over the next sections. In the former case, the low filler content of carbon nanotubes and soft elastomeric nature of PDMS can lead to minimal polymer reinforcement required to generate the desired piezoresistive behaviour. In the latter case, QTC and its unique and highly sensitive response under mechanical deformation was judged appropriate to evaluate the proposed sensor concept in the context of composites exhibiting either a positive or a negative piezoresistive coefficient.

**Figure 1:**
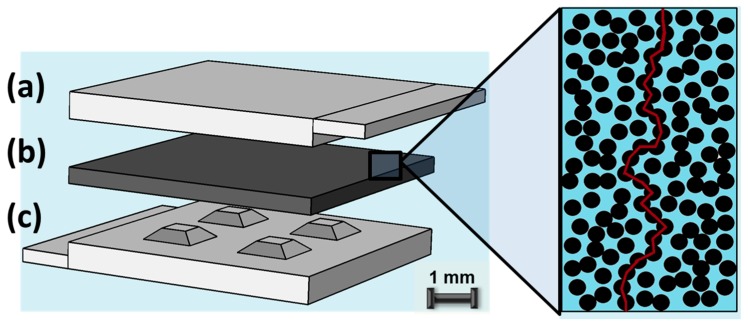
Schematic of a CNT-based elastomeric piezoresistive pressure sensor. Bottom and top layers (**a**,**c**) are the structured and unstructured aluminium electrodes, respectively, that encompass the piezoresistive filler composite (**b**), illustrated on the right. A direct conductive pathway is represented in this figure as a red curve.

As shown in Figure 1, the developed piezoresistive sensor incorporates the piezoresistive polymeric composite film in-between a microstructured and a flat electrode. Local pressure enhancement in the form of the microstructure truncated pyramidic features on the bottom electrode was implemented to enable a tuneable and improved sensor performance at lower operating pressure regimes. Both sensor types were characterized for compressive loads ranging from 50 kPa to 1.1 MPa. Section 2 presents the design and operating principle of both sensors, as well as the manufacturing process of the MWCNT-PDMS composite, device assembly and experimental setup of the measurement system. Results on the performance of the sensor are presented and discussed in Section 3. Section 4 concludes that the presented sensor approach results in an enhanced and tuneable resolution at lower pressure regimes.

## 2. Materials and Methods

### 2.1. Design of the Piezoresistive Sensor

The design of the piezoresistive sensor device, depicted in Figure 1, follows a simple three-layer configuration whereby two 5.25 × 5 mm^2^ aluminium electrodes, encapsulate a 300 μm thick piezoresistive filler-polymer composite film. The top and bottom aluminium layers, of thickness of 500 μm and 800 μm respectively, determine the piezoresistive response of the sensor to axial compressive loads only. The bottom electrode was patterned with a two-by-two array of equidistantly-spaced, 300 μm-high, truncated pyramidic structures. The surface area of these pyramids was varied from 75 × 75 μm^2^ to 550 × 550 μm^2^ to determine the relationship between sensor performance and surface area. Top and bottom aluminium contact pads, of equal thickness and length of 250 μm, protrude from the edges of the electrodes to enable electrical connection to an external bias voltage. The overall dimensions of the entire sensor are 5.5 × 5 × 1.6 mm^3^.

### 2.2. Composition of the Piezoresistive Composite Films

Two polymeric composite materials forming the sensing layer have been evaluated. The first material was developed in-house and is a nanocomposite consisting of MWCNT and PDMS. The second material utilised is a commercially available Quantum Tunnelling Composite (QTC) film (QTC “pill” product [20]) developed by the company Peratech (Brompton-on-Swale, UK) [21]. The material is used for example in single and multi-touch sensor configurations for applications spanning configurable force buttons to trackpads and under-display touch [21]. A similar formulation of such a composite has also been reported in [22]. The principles of conduction are quite different for these two materials, resulting respectively to a positive and a negative piezoresistive response under compressive deformation.

#### 2.2.1. CNT Based Nanocomposite Transduction

Conduction in CNT-based elastomeric composites emerges predominately due to the direct physical contact between neighbouring filler particles forming thereby continuous conducting pathways that span the composite (red curve, Figure 1) [23]. The transition from an insulating to a conductive phase occurs generally abruptly in such composites, with an exponential decrease in resistivity, at a critical filler concentration termed as the percolation threshold [23]. Such CNT-based composites exhibit an increase in resistivity when subjected to compressive deformation, unlike their conventional percolating composite counterparts that consist of low aspect ratio particles (spheroidal particles). In the latter case, compression leads primarily to the formation of new conductive pathways and hence to a decrease in resistivity, due to the very high percolation threshold (>30% by volume) that results in small average difference between fillers [24,25]. In contrast, during compression, CNTs experience bending which deteriorates their intrinsic electronic properties and significantly increases the resistivity of the composite as a consequence [12,26]. In addition, the material properties of the elastomeric matrix of the composite play an equally important role. An in-depth investigation and analysis of the above is described in [26]. Succinctly, CNT-based composites, in general, can exhibit both a negative piezoresistive response (NP), as well as, a positive one (PP) depending on the choice of the elastomeric matrix and its characteristic Poisson ratio *v*. The quantitative parameter that can be used to evaluate and determine the expected behaviour of these composites is the average junction gap variation (AJGV) between the filler particles, as adjacent CNTs are essentially electrically connected by the tunnelling transport of electrons through the junction gaps. AJGV in turn is effectively dominated by the Poisson ratio of the polymer constituent. For CNT-based composites with a Poisson ratio of the polymer matrix below a threshold (*v* ≤ 0.3) the composite exhibits a NP response during compressive deformation, as most junctions are either compressed or unchanged. However, when the Poisson ratio of the polymer is above the aforementioned threshold the composite starts to exhibit a PP response to compressive deformation. This is due to the complex counteracting relationship of junctions expanding and compressing, in combination with the interference of CNTs and the polymer chains which in turn serve as an obstacle to the junction gap change. In the case where the Poisson ratio of the polymer matrix is close to *v* = 0.5, such as PDMS, the aforementioned obstructing effect becomes dominant and the composite exhibits a purely NP response under compressive deformation. Hence, an increase of resistivity under compressive deformation is expected for such a pressure sensor.

#### 2.2.2. QTC Transduction Mechanism

QTC material was also evaluated as the piezoresisitive element of the sensor due its unique and highly sensitive piezoresistive response under mechanical deformation. The development of the composite material involves the uniform dispersion, into a polymeric silicone matrix, of micron-sized nickel particles with a spiky surface morphology into a polymeric silicon matrix [27]. A low energy dispersion method is employed during processing, which enables the complete encapsulation of the filler particles by the elastomeric matrix and leads to an electrically insulating response during equilibrium, even at filler loadings as high as 25% by volume. The physics governing the piezoresistive response of the QTC material differ substantially from conventional percolating composites and predominately stem from a quantum tunnelling effect affecting neighbouring filler particles of the composite [22,27,28]. During mechanical deformation, the protruding nano-spikes of adjacent nickel particles come into closer proximity enabling a charge flow due to the Fowler-Nordheim (FN) quantum tunnelling effect (cold emission of electrons), facilitated by the localised field enhancement at the extremities of the spikes [27]. Since the probability of quantum tunnelling depends exponentially on the width of the intervening potential barrier, the piezoresistive response of the composite exhibits an exponential drop in resistivity of as high as seven orders of magnitude under either compressive or even tensile deformation, contrary to the resistive increase that typical percolating composites exhibit [22,27].

### 2.3. Operating Principle of the Piezoresistive Sensor

For both types of composites, the micro-structured electrode morphology was designed to enable a local compression enhancement and therefore an improved performance at lower dynamic pressure ranges. The uniform compressive load, *P* = *F*/*A,* of a force *F* applied at the sensor surface *A*, is evenly distributed across the truncated pyramids of respective surface area *a.* The piezoresistive film composite experiences therefore an augmented effective load, *P_eff_*_,_ which imparts an enhanced deformation and hence a significant piezoresistive response such that:

(1) $$P_{eff}=k\cdot P=\frac{A}{N\cdot a}\cdot P$$

where *N* is the number of truncated pyramids and *k* = *A*/(*N·a*) is the pressure enhancement factor experienced by the piezoresistive film due to the microstructured surface of the electrode.

### 2.4. Manufacturing Process of the MWCNT-PDMS Composite

MWCNTs, with a 98% relative purity of carbon, 2.1 mL/g density, average length 3–6 μm and with outer and inner dimensions of 10 ± 1 nm and 4.5 ± 0.5 nm respectively (Sigma-Aldrich, St. Louis, MO, USA), were chosen as the filler constituent of the MWCNT-PDMS nanocomposite. Due to the high aspect ratio of the filler particles, such composites are used in this article as they have been found to exhibit a percolation threshold of 0.3% to 2.5% [26,29,30,31], which is significantly lower compared to that of spheroidal particles such as carbon black or metal particles where a concentration of as high as 26% *w*/*w* has been reported [32]. PDMS (Sylgard 184, Dow Corning, Midland, MI, USA) was chosen as the elastomeric matrix of the composite over other elastomers such as PMMA, PLLA or polycarbonate [33,34,35], due to its low Young’s modulus of 1.7 MPa, low cost, chemical inertness and biocompatibility [36,37]. The combination of a soft elastomeric matrix and low filler content minimizes the unwanted mechanical reinforcement effects of the composite, reported in the case of spheroidal filler particles, for example [25]. The low filler content and minimal polymer reinforcement enables therefore a quantifiable deformation, in terms of piezoresistive response, under low compressive loads. 

As described in detail in the next paragraph, the preparation process of the MWCNT/PDMS involves multiple dispersion steps utilising ultrasonication, an organic solvent and mechanical stirring as in [38,39,40,41,42,43], to achieve a uniform distribution of the MWCNT filler particles within the elastomeric matrix and facilitate an isotropic composite conductivity. Due to the strong inter-tube Van der Waals attractive forces, CNTs form together highly aggregated agglomerates in their natural state and hence external mechanical energy is required to separate these aggregates [44,45,46,47]. A 20 kHz frequency, 500 W programmable ultrasonic probe (Fisher Scientific) directly immersed in the solution was employed, as it could generate the required dispersion in minutes [29,43], in contrast to sonication baths that typically require hours to achieve the desired dispersion [14,30,48]. Furthermore, due to the highly viscous nature of PDMS, the organic solvent toluene (Sigma-Aldrich) was also required to assist the dispersion of the CNTs as it exhibits good solubility with this polymer [43,47,49,50] and can be easily extracted prior to crosslinking the composite. 

**Figure 2:**
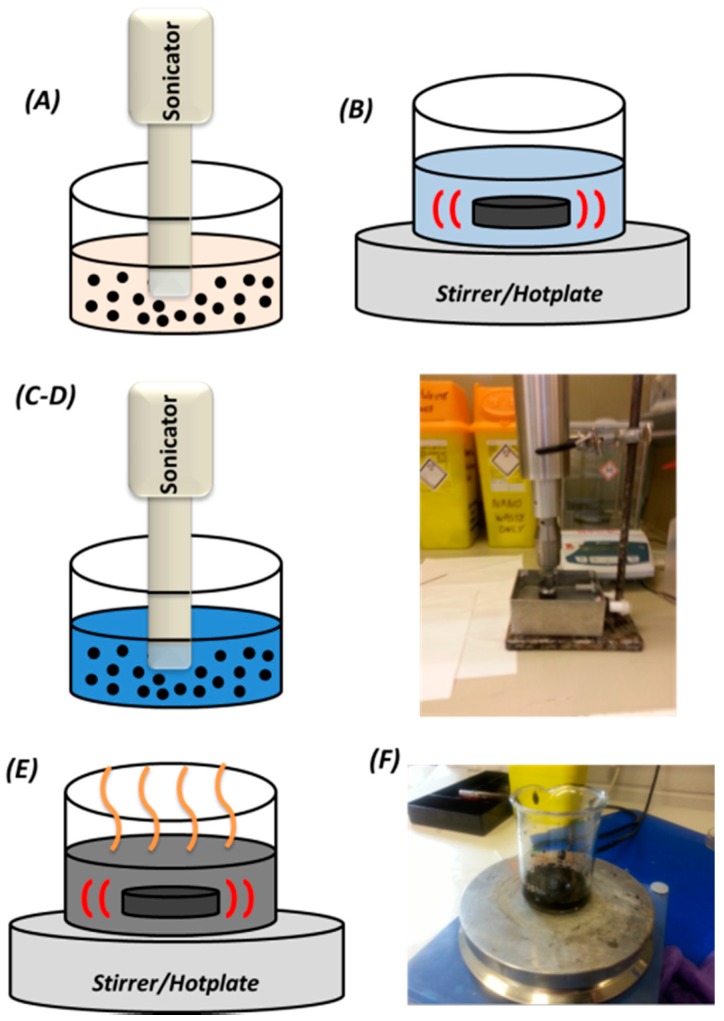
Preparation process of MWCNT/PDMS composite: (**A**) 1% *w*/*w* MWCNT/toluene solution ultrasonication; (**B**) shear mixing with magnetic stirrer of 10% *w*/*w* PDMS elastomer base/toluene; (**C**,**D**) combination of the two solutions to the desired filler ratio and ultrasonication; (**E**) shear mixing of solution at 100 °C to evaporate toluene; (**F**) addition of PDMS curing agent, shear mixing and degassing at desiccator prior to casting and thermal cross-linking of the composite films.

The procedure to manufacture the composite is presented in Figure 2. First, ultra-sonication of a 1% *w*/*w* MWCNTs/toluene solution in a pulse mode (10 s on, 15 s off) for 3 min at 20 kHz was implemented, to allow the solution to settle after each burst (A). Then 5 min shear mixing at 500 rpm of a 10% *w*/*w* PDMS elastomer base/toluene solution via a magnetic stirrer (B). The two solutions were combined at the desired filler-polymer ratio (C). The next step involves ultra-sonication for 3 min in a pulsed mode (10 s on, 15 s off) (D) and shear mixing via a magnetic stirrer at 100 °C and 500 rpm until the organic solvent had completely evaporated (45–60 min) (E). A high resolution digital balance (±0.01 g resolution) was utilised to monitor the evaporation rate, intermittently, as well as in all previous and subsequent process steps during development of the composite. Afterwards, the solution was cooled down to room temperature by immersing the vial into a cold water bath to avoid thermal crosslinking of the composite during the next step. Subsequently the curing agent of the Sylgard 184 PDMS was added to the solution, at the recommended 10:1 ratio [51] (F). The resulting composite was then shear mixed again for 15 min at 500 rpm with a magnetic stirrer, at room temperature. Finally, trapped air pockets were removed by placing the composite in a vacuum desiccator for 45 min. The composite was then cast in an aluminium mould with a 300 μm deep cavity and spread repeatedly via a blade to ensure an even distribution. The composite was thermally cross-linked at 100 °C for 1 h, and after demoulding, was then singulated to 5 × 5 mm^2^ blocks via a blade. The developed MWCNT/PDMS piezoresistive films were compared against 5 × 5 × 1 mm^3^ QTC films of the same structural configuration and under the same experimental conditions.

Structuring the electrode was achieved by a micromilling process, utilising a 1 mm diameter carbide mill tip (Roland MDX 20), which provided a feature accuracy of 25 μm. Aluminium was utilised as the structural material due its good conductivity, low cost and ease of milling. Multiple sets of the top unstructured and bottom structured electrode layers, with varying surface area of the truncated pyramid features, were produced from a single 1 mm thick aluminium 3 × 3 cm^2^ block via the use of laser-cutting. Sensors with truncated pyramids of 300 μm height and surface areas of 75 × 75 μm^2^, 200 × 200 μm^2^, 350 × 350 μm^2^, 425 × 425 μm^2^ and 550 × 550 μm^2^ were developed. The number of the pyramidic features was limited to four, solely due the diameter of the milling tip and the size of the sensor. The 300 μm high pyramidic geometry was chosen to ensure that any defects in the planarity of the micromilled areas outside the structures would not come into electrical contact with the piezoresistive film during compressive deformation, restricting thereby the response solely to the pyramidic features’ surface area.

**Figure 3:**
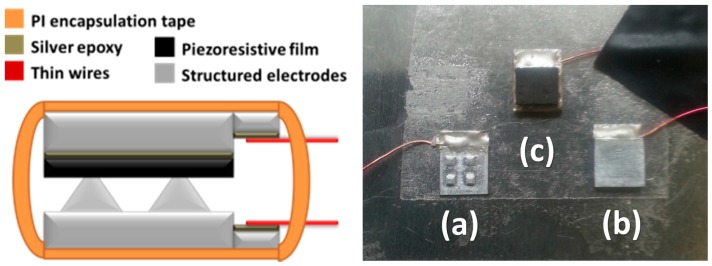
(**Left**) sensor assembly; (**right**) photograph of the individual parts of the sensor prior to encapsulation with (**a**) the structured electrode and (**b**,**c**) the unstructured electrodes with and without the piezoresistive film attached to it respectively.

The design of the proposed sensor enables a simple and cost-effective assembly as depicted in Figure 3. A thin layer of silver conductive epoxy was doctor-bladed on the surface of the flat electrode layer where the piezoresistive film was attached, ensuring maximum electrical contact after thermally curing the epoxy. Thin output wires were also similarly bonded to the electrode pads of both electrode layers to enable connection with a DC bias and the sensor configuration was finally set into place via a loose protective encapsulation with a thin polyimide (PI) tape.

### 2.5. Experimental Setup

**Figure 4:**
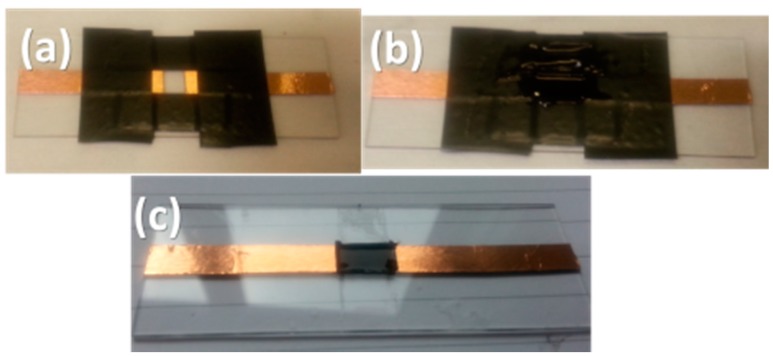
Experimental apparatus to characterise MWCNT/PDMS films: (**a**) prior to casting the composite; (**b**) composite casting; (**c**) cross-linked film.

A simple experimental apparatus was devised to extract the percolation threshold of the developed MWCNT/PDMS composite. As shown in Figure 4a, two copper tracks, placed 5 mm apart from each other, were laid out on a glass slide to define an area of 10 × 7 mm^2^. A small quantity of the composite was then poured and spread repeatedly on this area via a blade (Figure 4b), prior to curing, to form a film with a 300 ± 100 μm thickness as shown in Figure 4c.

**Figure 5:**
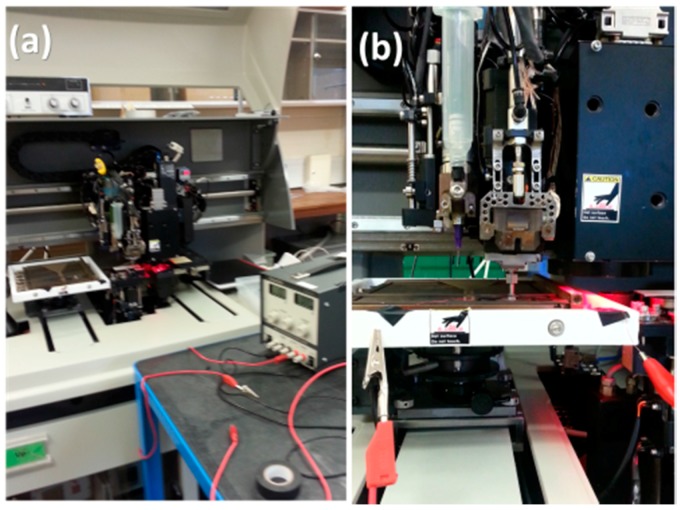
(**a**) MAT-400 die-bonder equipment and setup and (**b**) piezoresistive sensor during measurement.

The setup, powered by a DC supply of 1 V (TTi PL330 32 V-3 A PSU), was then connected in series to a Keithley 2000 digital multimeter to measure the resistance of MWCNT/PDMS films of various filler loadings ranging from 0.2% to 3%. Regarding the characterisation of the assembled piezoresistive sensors, detailed in Section 3, a MAT-400 die bonder (MAT, Yokneam Illit, Israel), shown in Figure 5, was employed to exert compressive loads from 40 kPa to 1.2 MPa. The bonder utilises a software-controlled probe with a micro-camera to ensure that uniform loads are accurately applied on the surface of the sensor within a user-set time limit (here 20 s).

## 3. Results and Discussion

### 3.1. MWCNT-PDMS Composite Characterisation

**Figure 6:**
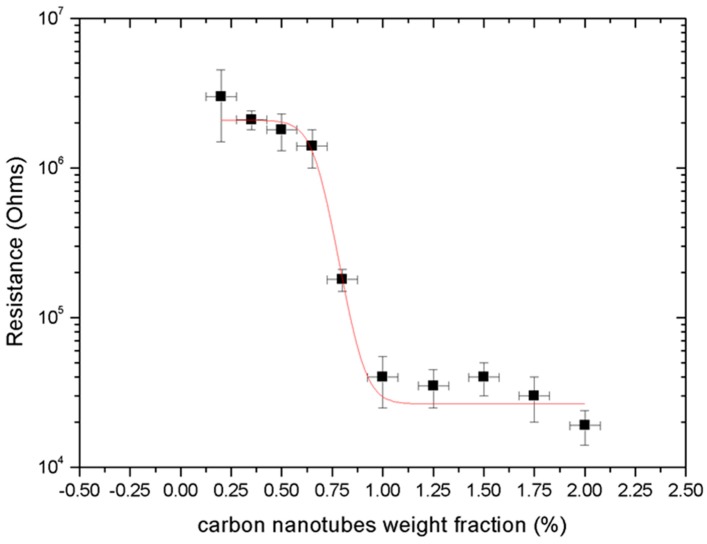
MWCNT/PDMS nanocomposite percolating behaviour.

As shown in Figure 6, the composite exhibits the typical sigmoidal conductive behaviour of percolating composites. A total of five composite batches were produced for each filler-polymer concentration displayed in Figure 6 and the composite was characterised with the experimental apparatus described in Section 2.5. The percolation threshold was found to be at a very low filler loading of approximately 0.625 ± 0.100% *w*/*w* that lies within the expected regime, as described previously. The resistance of the composite dropped significantly by close to three orders of magnitude from 5 MΩ to 18 kΩ, remaining relatively stable beyond the percolation threshold as expected from percolation theory [23]. 

This decrease in resistance was not as dramatic as reported in literature, where a change of as high as nine orders of magnitude has been documented [52,53]. A potential factor for the observed performance of the composite can be attributed to the extended shear mixing of the composite during the evaporation step which may have led to the fracturing of the CNTs and subsequent shortening of their length, resulting in a rather high value of the resistance of the composite beyond the percolation threshold, as reported elsewhere in [47,54]. 

Based on the results obtained in Figure 6, MWCNT/PDMS films were manufactured with a 0.75% filler ratio, which lies just beyond the percolation threshold. At this state, MWCNTs experience greater curvatures to compressive loading and hence intrinsically possess an enhanced piezoresistive response [12,24].

### 3.2. Characterisation of the Performance of the Sensors

#### 3.2.1. Characterisation of the MWCNT-PDMS Sensor

**Figure 7:**
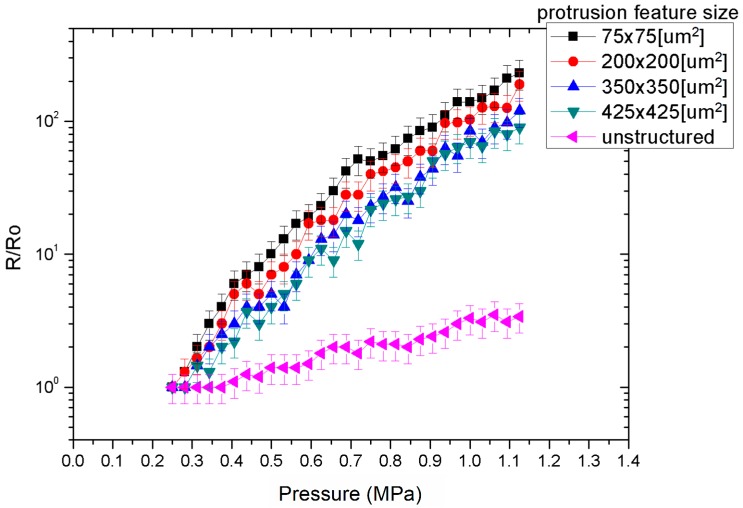
MWCNT/PDMS-based sensors performance vs. pyramidic features surface area.

The relative resistance of the MWCNT-PDMS sensor was characterised as a function of the compressive pressure load. A sensor with non-structured bottom electrode layer was first characterised as shown in Figure 7. Changes in relative resistance started appearing from pressure loads of approximately 0.45 MPa, with a 50% resistance increase at compressive loads as high as 1.15 MPa. Variations in pressure distinguishable from noise were recorded with a resolution of approximately 200 kPa. This response is in agreement with results reported in [12,13,14]. A notably improved response was observed when a microstructured bottom electrode was used. The larger dynamic range of the sensor enabled the capture of compressive loads of as low as 0.25 MPa, while the relative resistance increased significantly over the same pressure range. For example, a sensor with pyramidic features of 75 × 75 μm^2^ surface areas displayed a two-order increase over the initial resistance for loads as high as 1.15 MPa.

In this case pressure variations distinguishable from noise were extracted with a resolution as high as 50 kPa. For a given compressive pressure load, the relative resistance was also increased as the surface of area of the features decreased. For example, the relative resistance increased threefold between structures of 75 × 75 mm^2^ to 425 × 425 mm^2^ surface area at a compressive load of 650 kPa. 

#### 3.2.2. Characterisation of the QTC-Based Sensor

**Figure 8:**
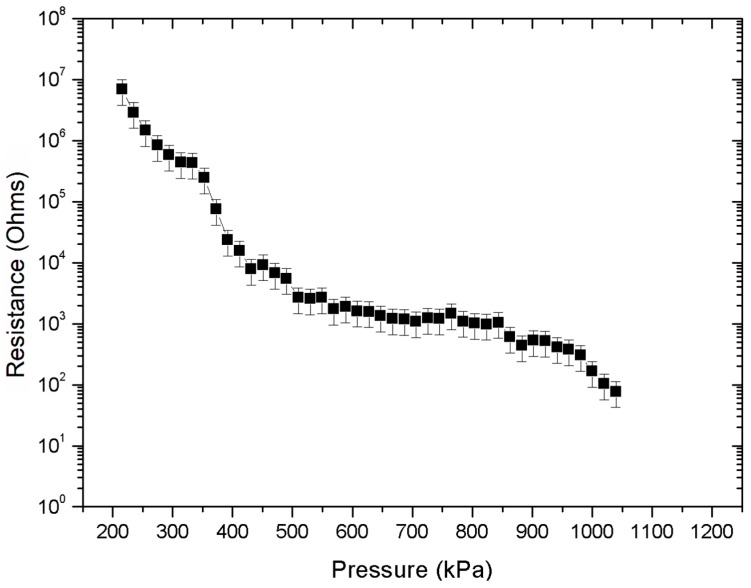
QTC pressure sensor performance with unstructured electrode layer.

Similar enhanced changes of relative resistance were also measured in the case of QTC-based sensors. As before, the response to compressive loading was first evaluated with an unstructured bottom electrode layer as shown in Figure 8.

QTC effectively behaved as an electrical insulator until a critical compressive load was applied as expected. Beyond that point, the resistance of the sensor decreased exponentially by five orders of magnitude from approximately 10 MΩ to less than 100 Ω for a pressure range of approximately 0.2 MPa to 1.1 MPa with a pressure resolution of as high as 50 kPa. The observed piezoresistive response of the QTC composite under compressive loads was in agreement with the results reported in [22,55].

**Figure 9:**
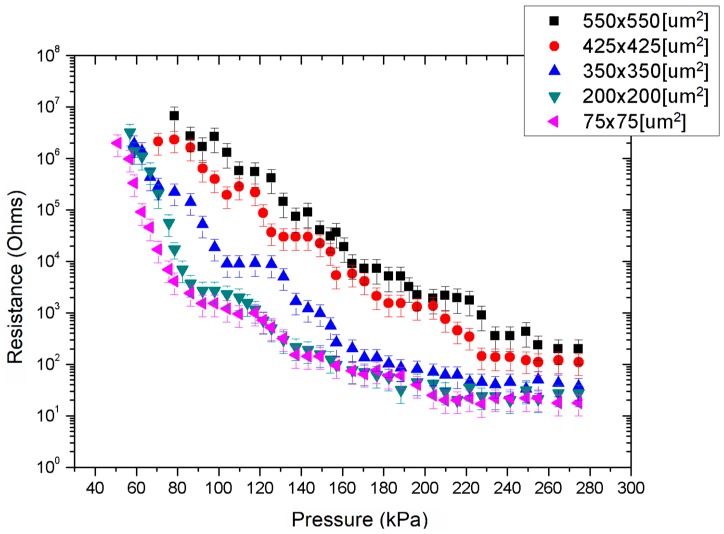
QTC-based sensors performance as a function of pyramidic microstructures surface area.

When truncated pyramids were introduced on the bottom electrode, the dynamic range and pressure resolution of the QTC-based sensor increased substantially, exhibiting a similar exponential response in a lower pressure regime as in the CNT-PDMS sensor, as shown in Figure 9. The smallest detectable pressure load in this case was approximately 100 kPa for a sensor with 550 × 550 μm^2^ surface area and 50 kPa for a sensor with 75 × 75 μm^2^ surface area. The resistance of the sensor exhibited a plateau at approximately 280 kPa, in the former case, to approximately 200 kPa in the latter. Pressure variations distinguishable from noise were recorded with a resolution of 10 kPa in this case. 

The microstructuring of electrodes therefore significantly improves the performance of both types of sensors. This patterning enables the detection of much smaller compressive loads with significantly enhanced resolution. Manipulation of the features dimensions also enables the effective tuning of the performance of the sensor in agreement the analysis of Section 2. 

Although the QTC-based sensor displayed a quantifiable response at a lower pressure regime and a higher pressure resolution, the variation of the resistance was strongly nonlinear with applied load. In contrast, the MWCNT-PDMS-based sensors displayed a more predictable response. The erratic response of the QTC-based sensors was translated as significant variations in the resistance values requiring a 10 to 20 s delay to reach a relatively stable resistance value prior to each pressure measurement.

**Figure 10:**
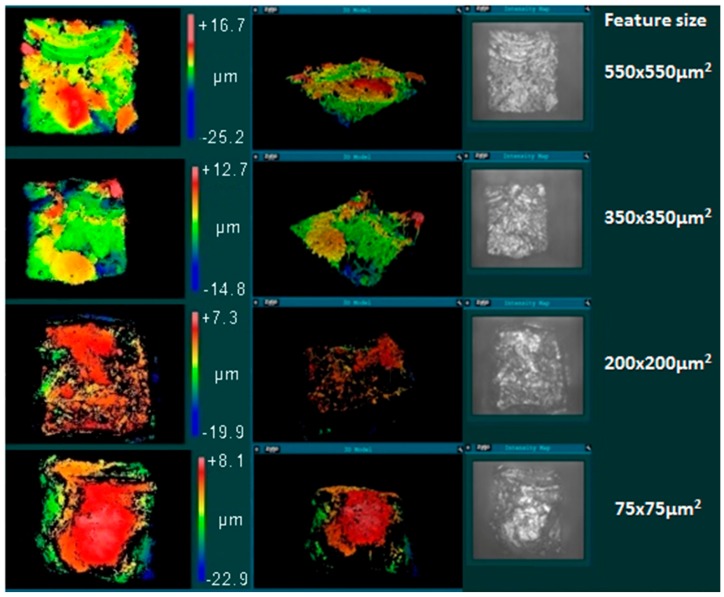
Morphology of pyramidic surface under optical profilometry with scale range and colour map on the left referring to the planarity of the surface.

Two reasons are possible regarding the observed nonlinear behaviour of the resistance as a function of the applied load. The first reason stems from the complex physical behaviour of QTC and MWCNT-PDMS materials when subject to a compressive load as alluded previously. Another reason relates to the non-uniform surface area of the pyramidic features generated during the micromilling process which led to an incomplete contact with the surface of the piezoresistive film and therefore a non-uniform deformation. The surface of the developed pyramidic features exhibited random small bumps and recesses, with an average height deviation in the order of approximately 10 μm. Furthermore, as the surface area decreased, the surface of the microstructured truncated pyramidic features deteriorated the square geometry into an almost circular shape as shown in the profilometric measurement in Figure 10 using a white light phase shifting interferometer (Zygo Viewmeter 4200), with the 75 × 75 μm^2^ surface area features displaying the least defined and uniform morphology. 

## 4. Conclusions

A small-sized piezoresistive pressure sensor was developed to measure low pressure ranges. The sensor makes use of a surface microstructured electrode with four symmetrically placed truncated pyramidic structures to enable an enhanced response in lower compressive regimes due to the reduced effective area of the exerted forces. A low cost manufacturing process was employed for the sensor development and assembly. Two different piezoresistive films were evaluated as the transducer layer of the sensor: a commercially available QTC film with a negative piezoresistive response and an in-house developed MWCNT-PDMS composite film with a positive piezoresistive response. 

Sensors based on a microstructured electrode displayed a significantly improved response and resolution in lower pressure regimes over sensors with their planar unstructured electrode counterparts. For the MWCNT-PDMS sensor, a resistance increase as high as two orders of magnitude was measured for loads up to 1.15 MPa between the structured and unstructured electrode type of sensor. Detection of pressure variations for compressive loads as low as 200 kPa with a resolution as high as 50 kPa respectively was achieved. In the case of the QTC-based sensor, loads as low as 50 kPa with a resolution increase of 10 kPa were measured for the microstructured electrode, as well as a reduced measurable range. Further improvement of the pressure resolution and increased tunability of the sensor performance are potentially feasible by utilizing structured electrodes for both the top and bottom layers of the sensor.

## References

[1] Chen W., Zhu Z., Tao X.M. (2015). Flexible Actuators. Handbook of Smart Textiles.

[2] Zang Y.P., Zhang F.J., Di C.A., Zhu D.B. (2015). Advances of flexible pressure sensors toward artificial intelligence and health care applications. Mater. Horiz..

[3] Liu H., Zhu L.L., He Y., Cheng B.W. (2017). A novel method for fabricating elastic conductive polyurethane filaments by in-situ reduction of polydopamine and electroless silver plating. Mater. Des..

[4] Deng H., Skipa T., Bilotti E., Zhang R., Lellinger D., Mezzo L., Fu Q., Alig I., Peijs T. (2010). Preparation of High-Performance Conductive Polymer Fibers through Morphological Control of Networks Formed by Nanofillers. Adv. Funct. Mater..

[5] Zhang H., Liu N.S., Shi Y.L., Liu W.J., Yue Y., Wang S.L., Ma Y.N., Wen L., Li L.Y., Long F. (2016). Piezoresistive Sensor with High Elasticity Based on 3D Hybrid Network of Sponge@CNTs@Ag NPs. ACS Appl. Mater. Interfaces.

[6] Stassi S., Cauda V., Canavese G., Pirri C.F. (2014). Flexible tactile sensing based on piezoresistive composites: A review. Sensors.

[7] Nabar B.P., Celik-Butler Z., Butler D.P. (2014). Self-Powered Tactile Pressure Sensors Using Ordered Crystalline ZnO Nanorods on Flexible Substrates Toward Robotic Skin and Garments. IEEE Sens. J..

[8] Zhu G., Yang W.Q., Zhang T., Jing Q., Chen J., Zhou Y.S., Bai P., Wang Z.L. (2014). Self-powered, ultrasensitive, flexible tactile sensors based on contact electrification. Nano Lett..

[9] Roberts P., Damian D.D., Shan W., Lu T., Majidi C. Soft-Matter Capacitive Sensor for Measuring Shear and Pressure Deformation. Proceedings of the IEEE International Conference on Robotics and Automation (ICRA).

[10] Mannsfeld S.C.B., Tee B.C.-K., Stoltenberg R.M., Chen C.V.H.-H., Barman S., Muir B.V.O., Sokolov A.N., Reese C., Bao Z. (2010). Highly sensitive flexible pressure sensors with microstructured rubber dielectric layers. Nat. Mater..

[11] Mitrakos V., Macintyre L., Denison F., Hands P., Desmulliez M.P.Y. (2017). Design, manufacture and testing of capacitive pressure sensors for low-pressure measurement ranges. Micromachines.

[12] Hu C.H., Liu C.H., Chen L.Z., Peng Y.C., Fan S.S. (2008). Resistance-pressure sensitivity and a mechanism study of multiwall carbon nanotube networks/poly(dimethylsiloxane) composites. Appl. Phys. Lett..

[13] Wang P., Geng S., Ding T. (2010). Effects of carboxyl radical on electrical resistance of multi-walled carbon nanotube filled silicone rubber composite under pressure. Compos. Sci. Technol..

[14] Khan S., Dahiya R., Tinku S., Lorenzelli L. (2014). Flexible tactile sensors using screen printed P(VDF-TrFE) and MWCNT/PDMS composites. IEEE Sens. J..

[15] Wang L., Peng H., Wang X., Chen X., Yang C., Yang B., Liu J. (2016). PDMS/MWCNT-based tactile sensor array with coplanar electrodes for crosstalk suppression. Microsyst. Nanoeng..

[16] Azkar S., Hasan U., Jung Y., Kim S., Jung C.-L., Oh S., Kim J., Lim H. (2016). A Sensitivity Enhanced MWCNT/PDMS Tactile Sensor Using Micropillars and Low Energy Ar+ Ion Beam Treatment. Sensors.

[17] Lee K., Lee S.S., Lee J.A., Lee K.-C., Ji S. (2010). Carbon nanotube film piezoresistors embedded in polymer membranes. Appl. Phys. Lett..

[18] Jung D., Lee K.H., Kim D., Burk D., Overzet L.J., Lee G.S. (2013). Highly Conductive Flexible Multi-Walled Carbon Nanotube Sheet Films for Transparent Touch Screen. Jpn. J. Appl. Phys..

[19] Weng X., Mark G.A. Fabrication of patterned carbon nanotube (CNT)/elastomer bilayer material and its utilization as force sensors. Proceedings of the TRANSDUCERS 2009 International Solid-State Sensors, Actuators and Microsystems Conference.

[20] Lascells. http://www.lascells.com/products/product.php?s=qtc-pills.

[21] Peratech. https://www.peratech.com/.

[22] Canavesea G., Stassia S., Fallautoc C., Corbellinic S., Caudaa V., Camarchiaa V., Pirolac M., Pirria C.F. (2014). Piezoresistive flexible composite for robotic tactile applications. Sens. Actuators A Phys..

[23] Zhang W., Dehghani-Sanij A.A., Blackburn R.S. (2007). Carbon based conductive polymer composites. J. Mater. Sci..

[24] Dang Z.M., Jiang M.J., Xie D., Yao S.H., Zhang L.Q., Bai J. (2008). Supersensitive linear piezoresistive property in carbon nanotubes/silicone rubber nanocomposites. J. Appl. Phys..

[25] Hussain M., Choa Y.H., Niihara K. (2001). Conductive rubber materials for pressure sensors. J. Mater. Sci. Lett..

[26] Wang Z., Ye X. (2014). An investigation on piezoresistive behaviour of carbon nanotube/polymer composites: II. Positive piezoresistive effect. Nanotechnology.

[27] Bloor D., Donnelly K., Hands P.J., Laughlin P., Lussey D. (2005). A metal polymer composite with unusual properties. J. Phys. D Appl. Phys..

[28] Hands P.J.W. (2003). Vapour Sensing Applications and Electrical Conduction Mechanisms of a Novel Metal-Polymer Composite. Ph.D. Thesis.

[29] Jang S.H., YinBath H.M. (2015). Effect of aligned ferromagnetic particles on strain sensitivity of multi-walled carbon nanotube/polydimethylsiloxane sensors. Appl. Phys. Lett..

[30] Sagar S., Iqbal N., Maqsood A. (2013). Dielectric, electric and thermal properties of carboxylic functionalized multiwalled carbon nanotubes impregnated polydimethylsiloxane nanocomposite. J. Phys. Conf. Ser..

[31] Bauhofer W., Kovacs J.Z. (2009). A Review and Analysis of Electrical Percolation in Carbon Nanotube Polymer Composites. Compos. Sci. Technol..

[32] Niu X.Z., Peng S.L., Liu L.Y., Wen W.J., Sheng P. (2007). Characterizing and patterning of PDMS-Based conducting composites. Adv. Mater..

[33] Kang I., Schulz M.J., Kim J.H., Shanov V., Shi D. (2006). A Carbon Nanotube Strain Sensor for Structural Health Monitoring. Smart Mater. Struct..

[34] Liu Y., Chakrabartty S., Gkinosatis D.S., Mohanty A.K., Lajnef N. Multi-walled Carbon Nanotubes/Poly(L-lactide) Nanocomposite Strain Sensor for Biomechanical Implants. Proceedings of the Biomedical Circuits and Systems Conference.

[35] Zhang W., Suhr J., Koratkar N. (2006). Carbon Nanotube/Polycarbonate Composites as Multifunctional Strain Sensors. J. Nanosci. Nanotechnol..

[36] Charitidis C.A. (2011). Nanoscale Deformation and Nanomechanical Properties of Polydimethylsiloxane (PDMS). Ind. Eng. Chem. Res..

[37] Lotters J.C., Olthuis W., Veltink P.H., Bergveld P. (1997). The Mechanical Properties of the Rubber Elastic Polymer Polydimethylsiloxane for Sensor Applications. J. Micromech. Microeng..

[38] Liu B., Chen Y., Luo Z., Zhang W., Tu Q., Jin X. (2015). A novel method of fabricating carbon nanotubes-polydimethylsiloxane composite electrodes for electrocardiography. J. Biomater. Sci. Polym. Ed..

[39] Sun K., Xie P., Wang Z., Su T., Shao Q., Ryu J., Zhang X., Guo J., Shankar A., Li J. (2017). Flexible polydimethylsiloxane/multi-walled carbon nanotubes membranous metacomposites with negative permittivity. Polymer.

[40] Cui J., Zhang B., Duan J., Guo H., Tang J. (2016). Flexible Pressure Sensor with Ag Wrinkled Electrodes Based on PDMS Substrate. Sensors.

[41] Manzoor M.U., Lemoine P., Dixon D., Hamilton J.W.J., Maguire P.D. (2015). Stretchable conducting gold films prepared with composite MWNT/PDMS substrates. AIP Adv..

[42] Mazlan N., Jaffar M., Aziz A., Ismail H., Busfiled J.J.C. (2016). Effects of different processing techniques on multiwalled carbon nanotubes/silicone rubber nanocomposite on tensile strength properties. IOP Conf. Ser. Mater. Sci. Eng..

[43] Khosla A., Gray B.L. (2012). Micropatternable Multifunctional Nanocomposite Polymers for Flexible Soft NEMS and MEMS Applications. ECS Trans..

[44] Kim Y.A., Hayashi T., Fukai Y., Endo M., Yanagisawa T., Dresselhaus M.S. (2002). Effect of Ball Milling on Morphology of Cup-Stacked Carbon Nanotubes. Chem. Phys. Lett..

[45] Inkyo M., Tahara T., Iwaki T., Iskandar F., Hogan C.J., Okuyama K. (2006). Experimental Investigation of Nanoparticle Dispersion by Beads Milling with Centrifugal Bead Separation. J. Colloid Interface Sci..

[46] Sato H., Sano M. (2008). Characteristics of Ultrasonic Dispersion of Carbon Nanotubes Aided by Antifoam. Colloids Surf. A Physicochem. Eng. Asp..

[47] Huangm Y.Y., Terentjev E.M. (2012). Dispersion of Carbon Nanotubes: Mixing, Sonication, Stabilization, and Composite Properties. Polymers.

[48] Caneba G.T., Dutta C., Agrawal V., Rao M. (2010). Novel Ultrasonic Dispersion of Carbon Nanotubes. J. Miner. Mater. Charact. Eng..

[49] Liu C.-X., Choi J.-W. (2012). Improved Dispersion of Carbon Nanotubes in Polymers at High Concentrations. Nanomaterials.

[50] Cavaco A., Ramalho A., Pais S., Durães L. CNT-Polydimethylsiloxane nanocomposites for prosthesis interfaces. Proceedings of the 10th International Conference on Composite Science and Technology.

[51] Dow Corning. http://www.dowcorning.com/DataFiles/090276fe80190b08.pdf.

[52] Vilčáková J., Moučka R., Svoboda P., Ilčíková M., Hřibová N.K.M., Mičušík M., Omastová M. (2012). Effect of Surfactants and Manufacturing Methods on the Electrical and Thermal Conductivity of Carbon Nanotube/Silicone Composites. Molecules.

[53] Huang Y.Y., Marshall J., Gonzalez-Lopez C., Terentjev E.M. (2011). Variation in carbon nanotube polymer composite conductivity from the effects of processing, dispersion, aging and sample size. Mater. Express.

[54] Hwang J., Jang J., Hong K., Kim K.N., Han J.H., Shin K., Park C.E. (2011). Poly(3-hexylthiophene) Wrapped Carbon Nanotube/Poly(Dimethylsiloxane) Composites for use in Finger-Sensing Piezoresistive Pressure Sensors. Carbon.

[55] Graham A. (2008). Electrical Properties and Vapour Sensing Characteristics of a Novel Metal-Polymer Composite. Ph.D. Thesis.

